# Effects of Peritonsillar Injection of Tramadol and Adrenaline before Tonsillectomy

**Published:** 2013-06

**Authors:** Zafarullah Beigh, Mudasir ul Islam, Shakil Ahmad, Rafiq Ahmad Pampori

**Affiliations:** 1 *Department of Otorhinolaryngology. Govt. Medical College Srinagar J&K India. Srinagar. India.*

**Keywords:** Adrenaline, Heamostasis, Pain, Tonsillectomy, Tramadol

## Abstract

**Introduction:**

Various hemostatic and analgesic agents and techniques have been used to reduce intraoperative and postoperative hemorrhage and pain in tonsillectomy.Aims and objective; The current study aimed to compare the effect of using adrenaline plus tramadol and normal saline in maintaining hemostasis and control of pain in cold dissection tonsillectomy.

**Materials and Methods:**

This prospective randomized study was conducted over a period of 10 months in department of otorhinolaryngology state medical college Srinagar. In the current study 46 patients planed for tonsillectomy were put into two groups. 23 patients in each group. In group A patients (study group) 4ml of solution containing 1:200000 adrenaline and 2mg/kg tramadol was injected in peritonsillar space. In group B patients (control group) 4ml of normal saline was injected in peritonsillar space.

**Results:**

It was found that the time required to achieve heamostasis and post operative pain was less in group A patients in comparison to Group B patients. There was no significant side effect or complications when adrenaline and tramadol were used.

**Conclusion:**

Large randomized controlled studies are needed to compare tramadol plus adrenaline infiltration with other heamostatic and analgesics, but the current study indicated that Tramadol plus adrenaline infiltration could be an effective method to reduce the post operative pain , operative time and time to achieve heamostasis in tonsillectomy surgeries. Therefore the use of Tramadol plus adrenaline infiltration should be further promoted and implemented as routine use in tonsillectomy surgeries.

## Introduction

Tonsillectomy with or without adenoid- ectomy is one of the most frequent performed procedures in ENT departments. Frequently, tonsillectomies are associated with significant periop-erative bleeding and postoperative pain (1). Peritonsillar injection of various local anesthetics with addition of adrenaline in order to reduce posttonsillectomy pain and perioperative bleeding is one of the strategies which have been developed (2). Lidocaine–adrenaline combination is commonly used for infiltrative anesthesia because of its rapid action and providing some degree of hemostasis (3).

Hemorrhage is one of the most important complications of tonsillectomy which could be primary, reactionary or secondary. To avoid this complication, proper preoperative preparation of patients is essential by proper history taking, clinical examination and investigations to rule out bleeding disorders in patients and to ensure that patients are optimized for the procedure.

Controversies exist in the preoperative evaluation of patients for tonsillectomy especially regarding hemorrhage and its control (Bolger et al., 1990; Gabriel et al., 2000; Asaf et al., 2001) but this paper would not dwell on these issues.

The most important primary cause for morbidity following tonsillectomy in early postoperative period is Pain. This results in decrease of oral intake, dehydration and also prolonged hospitalisation. Various methods of decreasing postoperative pain have been devised. Boliston and Upton(4) showed that the local infiltration of lidnocaine 0.5% containing epinephrine into peritonsillar bed of adults undergoing tonsillectomies under general anesthesia resulted in greater ease in dissection as well as significant reduction in operative blood loss. Ginstrom et al (5) reported that intraoperative infiltration of bupivacaine/epinephrine had only marginal effect on pain. In a paediatric population, Naja et al (6) found that a preincision injection of local anesthesia resulted in significantly less postoperative pain at rest, on jaw opening and during intake of a soft diet compared with findings in a placebo group.

Tramadol is a centrally-acting drug, which is effective in the treatment of moderate to severe pain (7). In addition to its systemic action, the local anesthetic effect of tramadol on peripheral nerves has been shown in both laboratory and clinical studies (8,9).

The current study aimed to evaluate the effect of peritonsillar infiltration when performed with one of the two different solution (Tramadol containing epinephrine and normal saline) upon time reduction in achieving complete heamostasis and postoperative pain levels

## Materials and Methods

After Institutional Ethics Committee approval and written informed consent was obtained from patients and/or their parents, 46 patients aged between 10 and 24 years, of ASA 1–2 status undergoing elective tonsillectomy were enrolled in a randomized double blind study. Patients having cardiac, pulmonary, hematologic or hepatic diseases, or known allergy to administered drugs, and the ones who had received any analgesic drug in the last 24 h were excluded from the study.

All procedures were performed under general anesthesia by a standard anesthetic protocol. Anesthesia was induced with 2 mg kg _1 propofol intravenously . After having an i.v. line inserted on the dorsum of the hand, infusion of the lactated ringer’s solution started. Following muscle relaxation with atracurium and tracheal intubation, anesthesia was maintained with isoflurane 2–3% and 50% nitrous oxide in oxygen. After the induction i.v paracetamol 5 mg kg _1 were given as infusion to all patients. After the proper positioning, children were randomly allocated into two groups using a shuffled, sealed, opaque and numbered envelopes to receive 8ml of tramadol plus 1:200000 adrenaline (group A, N=23), or 8 ml of saline (group B, N=30) preinsicionally. The trial was undertaken in a double blind manner, neither patients nor parents and the surgeons were aware of the injected drugs. The drugs were prepared into the injector making totally 8 ml by the anesthesiologist who was not included in the peri and postoperative evaluation and the injections were performed by the two senior surgeons each were blinded to the injected drug with 23 G needle using aspiration-injection technique. A total of 8 ml, 4 ml for each tonsil was injected into the pericapsular plane as follows: 1.5 ml into the superior pole, 1 ml in between and 1.5 ml into the lower pole. The operation was started with Mucosal incisions performed with cold knife and for tonsillectomy blunt dissection technical was used. Hemostasis was achieved by bi-polar cauterization. After removing the first tonsil a saline soaked gauze applied to surgical field. After removing the second tonsil, hemostasis was achieved by bipolar cauterization if needed. At the end of the surgery , number of the cautery used, time to achieve hemostasis (time registered use of swabs and diathermy), and operation duration was measured. All patients were reversed from anaesthesia using 0.02 mg kg _1 atropin and 0.05 mg kg _1 neostigmine. After the airway reflexes were secured patients were extubated and the children were transferred to PACU(post Anaesthesia care unit) for 30 min follow up.

All patients were instructed preoperatively to use 100 mm Visual Analogue Scale (VAS) for pain (0 = no pain to 100 = the worst pain). The pain assessments started after 30 mins of operation and continued in the ward every hour for 6 hours postoperatively.

Postoperative supplementary analgesic was available to the patients in the form of inj diclofenac 1.5mg per kg was administered i.m if the recorded VAS score was 50 or greater. Bearable pain period of time was considered as the time from full recovery to the first requirement of diclofenac. The total diclofenac in mg per kg consumption during the first 6 hours was recorded. No other analgesics were administered.

The occurrence of side effects such as hypotension, bradycardia, nausea, vomiting, sedation, or other side effects was recorded for each patient at the same timepoints as defined for VAS assessment.

A total of 46 patients completed the study. Demographic data (age, gender, weight and ASA) of all subjects were similar in both groups (Table. 1). 

Time required for hemostasis, the number of cautery used for hemostasis and operation duration were significantly lower in group A compared to group B (P<0.001), (Table.2). Post operative visval analogue pain score was less in group A in comparison to Group B (P<0.005), (Table.3), also post operative supplemental analgesic required in Group A patients was very less in comparison to Group B (<0.005) (Table. 4).

**Table 1:** Demographic Data

Variable	GroupA n=23	GroupB n=23	P value
Age (Y)	17+4	16+5	NS
Gender (M/F)	13/10	14/9	NS
Weight (kg)	40+10	38+9	NS
ASA (I/II)	17/6	19/4	NS

The data were in mean+SD

ASA (American society of anaesthesio-logists) classification of physical status.

**Table 2:** Operative Data

Variables	Group A	Group B	P value
Heamostasis time (min)	8+ 2	15+3	<0.001
No.of cautery used	3+2	7+3	<0.001
Operation time	15+4	25+5	<0.001

The data were in mean + SD

**Table 3:** Post operative visval analogue pain score comparison

**Time after** **surgery**	**VAS pain score ** **in Group A**	**VAS pain score in Group B**	**P value**	
30 min	30+10	70+10	<0.005
1 hour	30+10	70+10	<0.005
2 hours	30+10	60+10	<0.005
3 hours	40+10	60+10	<0.005
4 hours	40+10	60+10	<0.005
5 hours	45+10	55+10	<0.005
6 hours	45+10	55+10	<0.005

Data were in mean + SD

**Table 4:** Comparison of post operative diclofenac used in both groups

	Group A	Group B	P value
Amount of inj diclofenac used in post operative period (mg/kg)	0mg/kg	2+1mg/kg	<0.005

There were no significant side effects such as hypotension, bradycardia, nausea, vomiting, sedation, or other side effects in study group in comparison to control group.

## Discussion

Postoperative pain and its sequelae are amongst universal complaints of the patients, and post-tonsillectomy pain remains a considerable clinical problem.

The current study showed that pre-tonsillectomy infiltration of tramadol 2 mg× kg- (4 ml per tonsil); containing 1/200,000 adrenalin, reduces immediate postoperative pain in patients compared with placebo.

The findings are in agreement with results from a previous study (10), where peritonsillar infiltration of tramadol in pediatric patients provided superior postoperative analgesia to placebo for 4 hours after surgery. In addition, in that study, tramadol group received significantly more doses of paracetamol than placebo group in order to maintain analgesia in the first 12 hours after recovery from anesthesia (10).

Tramadol was thought to produce its antinoceptive and analgesic effects through spinal and supraspinal sites rather than via a local anesthetic action. However, several clinical studies have shown that tramadol might have peripheral local anesthetic effect (11,12). By direct tramadol application to the sciatic nerve in rats, it was proven that tramadol exerts a local anesthetic effect (13).

In the present study, tramadol had a local anesthetic action similar to that of lidocaine, and because of its antinociceptive effect, it could extend the postoperative pain-free period.

When extracellular sodium concentration decreases, the nerve fiber becomes sensitive to local anesthetics (14). Jou et al, (15) suggested that tramadol affects sensory and motor nerve conduction by a similar mechanism to that of lidocaine, which acts on the voltage-dependent sodium channel leading to axonal blockage. However, Mert et al, proposed that tramadol might have a mechanism different from that of lidocaine for producing conduction blocks (16); the presence of a large Ca+2 concentration in the external medium increases tramadol's activity whereas decreasing lidocaine's activity.

Akbay et al, studied the effects of topical tramadol on postoperative pain and morbidity in children undergoing tonsillec- tomy (17), and concluded that topical 5% tramadol with its local anesthetic effect seems to be an easy, safe, and comfortable approach for pain management in children undergoing tonsillectomy.

In another study to investigate the efficacy of intramuscular injection and peritonsillar infiltration of tramadol to prevent pain in children undergoing tonsillectomy (18), it was concluded that peritonsillar infiltration with tramadol provided good intraoperative analgesia, less postoperative pain on awakening and lower analgesic requirement within the first hour after surgery.

Nausea and vomiting have been major side effects of tramadol used as postoperative analgesia. The incidence of these side effects seems to be mainly related to the peak serum concentrations, an initial IV loading dose of 3 mg/kg caused more symptoms than a subsequent infusion or patient-controlled analgesia (19).

In a study comparing the postoperative analgesic efficacy and side effects of IV tramadol with peritonsillar infiltration of tramadol in children undergoing adenotonsi- llectomy (20), it has been demonstrated that peritonsillar infiltration of tramadol maintains efficient pain relief with lower incidence of nausea and vomiting in comparison to IV administration. It was concluded that peritonsillar infiltration of tramadol 2mg×kg-1 provides post-tonsillectomy pain control in the first 6 postoperative hours compared to that of normal saline Hemorrhage is a frequent and often the most dreaded complication of tonsillectomy which has prompted the use of a variety of topical haemostatic agents such as bismuth subgallate and adrenaline to achieve hemostasis and reduce the risk of intra and postoperative hemorrhage (Rasgon et al., 1991; Agrawal et al., 2005; Sharma et al., 2007).

Although the conventional techniques to secure hemostasis like ligation and/or cauterization control the major bleeders yet occasionally the diffuse bleeding and capillary oozing may pose difficulty for the surgeon to locate the bleeders, contribute to inefficient hemostasis and in turn enhance blood loss and prolong operation time (Callanan et al., 1995). Adrenaline which is the commonly used topical hemostatic agent in our region is a powerful vaso-constrictor producing retraction of blood vessels in the tonsillar bed. Adrenaline also promotes platelet aggregation in the formation of blood clot and has been demonstrated by Hatton to be inexpensive, posing little risk, and decreasing intraoperative bleeding, therefore can be taken as a reasonable hemostatic agent (Hatton,2000).These characteristics especially the decrease in intraoperative bleeding and invariably decrease in intraoperative time is demonstrated in the current study. Less intra operative bleeding leads to less use of cautery to achieve heamostasis and it added to comparatively less post operative pain in groupA patients in the current study.

## Conclusion

Large randomized controlled studies are needed to compare tramadol plus adrenaline infiltration with other heamostatic and analgesics, but the current study indicated that Tramadol plus adrenaline infiltration could be an effective method to reduce the post operative pain , operative time and time to achieve heamostasis in tonsillectomy surgeries. Therefore the use of Tramadol plus adrenaline infiltration should be further promoted and implemented as routine use in tonsillectomy surgeries.
